# Genetic and Non-genetic Predictors of LINE-1 Methylation in Leukocyte DNA

**DOI:** 10.1289/ehp.1206068

**Published:** 2013-04-03

**Authors:** Salman M. Tajuddin, André F. S. Amaral, Agustín F. Fernández, Sandra Rodríguez-Rodero, Ramón María Rodríguez, Lee E. Moore, Adonina Tardón, Alfredo Carrato, Montserrat García-Closas, Debra T. Silverman, Brian P. Jackson, Reina García-Closas, Ashley L. Cook, Kenneth P. Cantor, Stephen Chanock, Manolis Kogevinas, Nathaniel Rothman, Francisco X. Real, Mario F. Fraga, Núria Malats, for the Spanish Bladder Cancer/EPICURO Study Investigators

**Affiliations:** 1 Genetic and Molecular Epidemiology Group, Spanish National Cancer Research Centre (CNIO), Madrid, Spain; 2 Cancer Epigenetics Laboratory, Instituto Universitario de Oncología del Principado de Asturias (IUOPA), Oviedo, Spain; 3 National Cancer Institute, National Institutes of Health, Department of Health and Human Services, Bethesda, Maryland, USA; 4 Universidad de Oviedo, Oviedo, Spain; 5 Hospital General Universitario de Elche, Elche, Spain; 6 Hospital Ramon y Cajal, Madrid, Spain; 7 Division of Genetics and Epidemiology, Institute of Cancer Research, Sutton, UK; 8 Trace Element Analysis Core, Department of Earth Sciences, Dartmouth College, Hanover, New Hampshire, USA; 9 Unidad de Investigación, Hospital Universitario de Canarias, La Laguna, Spain; 10 KP Cantor Environmental LLC, Silver Spring, Maryland, USA; 11 Centre for Research in Environmental Epidemiology (CREAL), Barcelona, Spain; 12 Epithelial Carcinogenesis Group, Spanish National Cancer Research Centre (CNIO), Madrid, Spain; 13 Departament de Ciències Experimentals i de la Salut, Universitat Pompeu Fabra, Barcelona, Spain; 14 Department of Immunology and Oncology, Centro Nacional de Biotecnología/CNB-CSIC, Cantoblanco, Madrid, Spain

**Keywords:** DNA methylation, epigenetics, LINE-1, one-carbon metabolism gene variants, smoking, trace elements

## Abstract

Background: Altered DNA methylation has been associated with various diseases.

Objective: We evaluated the association between levels of methylation in leukocyte DNA at long interspersed nuclear element 1 (LINE-1) and genetic and non-genetic characteristics of 892 control participants from the Spanish Bladder Cancer/EPICURO study.

Methods: We determined LINE-1 methylation levels by pyrosequencing. Individual data included demographics, smoking status, nutrient intake, toenail concentrations of 12 trace elements, xenobiotic metabolism gene variants, and 515 polymorphisms among 24 genes in the one-carbon metabolism pathway. To assess the association between LINE-1 methylation levels (percentage of methylated cytosines) and potential determinants, we estimated beta coefficients (βs) by robust linear regression.

Results: Women had lower levels of LINE-1 methylation than men (β = –0.7, *p* = 0.02). Persons who smoked blond tobacco showed lower methylation than nonsmokers (β = –0.7, *p* = 0.03). Arsenic toenail concentration was inversely associated with LINE-1 methylation (β = –3.6, *p* = 0.003). By contrast, iron (β = 0.002, *p* = 0.009) and nickel (β = 0.02, *p* = 0.004) were positively associated with LINE-1 methylation. Single nucleotide polymorphisms (SNPs) in *DNMT3A* (rs7581217-per allele, β = 0.3, *p* = 0.002), *TCN2* (rs9606756-GG, β = 1.9, *p* = 0.008; rs4820887-AA, β = 4.0, *p* = 4.8 × 10^–7^; rs9621049-TT, β = 4.2, *p* = 4.7 × 10^–9^), *AS3MT* (rs7085104-GG, β = 0.7, *p* = 0.001), *SLC19A1* (rs914238, TC vs. TT: β = 0.5 and CC vs. TT: β = –0.3, global *p* = 0.0007) and *MTHFS* (rs1380642, CT vs. CC: β = 0.3 and TT vs. CC; β = –0.8, global *p* = 0.05) were associated with LINE-1 methylation.

Conclusions: We identified several characteristics, environmental factors, and common genetic variants that predicted DNA methylation among study participants.

DNA methylation plays a fundamental role in regulation of gene expression, genomic imprinting, X-chromosome inactivation, and repression of transposable elements (Jones and Liang 2009). Aberrant DNA methylation has been associated with various cancers and with developmental, autoimmune, and other chronic diseases (Robertson 2005). Global DNA methylation can be directly quantified by measuring 5-methylcytosine content of the genome, or it can be estimated based on methylation of repetitive sequences such as Alu elements or long interspersed nuclear element 1 (LINE-1) (Yang et al. 2004). Age, sex, smoking, and arsenic and lead exposures have been associated with DNA methylation, but findings have been inconsistent among studies (Breitling et al. 2011; El-Maarri et al. 2007; Fraga et al. 2005; Fuke et al. 2004; Terry et al. 2011). The folate and methionine-dependent one-carbon metabolism pathway could modulate DNA methylation by altering the level of *S*-adenosylmethionine (SAM), the principal source of methyl groups (Ulrey et al. 2005). Genetic variants might also influence the methylation of CpG loci locally, or might have a global influence on methylation throughout the genome. For example, a single nucleotide polymorphism (SNP) in *TRPC3* (transient receptor potential cation channel, subfamily C, member 3)-isoform 2 has been reported to regulate the methylation status of its own promoter (Martin-Trujillo et al. 2011), and variants of the methylenetetrahydrofolate reductase gene *(MTHFR)* have been associated with global DNA hypomethylation (Castro et al. 2004; Friso et al. 2002). However, although the determinants of global and site-specific methylation are widely assumed to be likely contributors to health and disease, they are poorly defined at this time.

Assessing the impact of both genetic and non-genetic factors on global DNA methylation may improve our understanding of the molecular pathogenesis of many common diseases. Therefore, we investigated associations of global DNA methylation in LINE-1 from bisulfite-modified granulocyte DNA with genetic variants and personal, demographic, lifestyle, and environmental characteristics.

## Methods

*Study population*. The study population, design, and data collection have been previously described (García-Closas M et al. 2005). Briefly, participating individuals were controls from the Spanish Bladder Cancer/EPICURO study who were admitted to hospitals in five regions of Spain for a range of conditions including hernia, fractures, and other noncancer diseases, and were 20–81 years of age. We collected demographic and exposure information at the hospitals using computer-assisted personal interviews. Of a total of 1,271 controls who agreed to participate in the study and were interviewed, 1,056 provided blood for DNA extraction. We excluded 23 subjects because of inadequate or poor quality DNA (*n* = 15) or missing smoking status data (*n* = 8). Three subjects with missing data on smoking status were included because they had data on other variables including age, sex, region, and body mass index (BMI). To ensure homogeneity, we also excluded one non-Caucasian individual, leaving 925 individuals with granulocyte DNA for bisulfite modification and pyrosequencing. Pyrosequencing failed in 33 individuals; thus, the final study population for the present analysis included 892 participants. We obtained written informed consent from all participants, and the study was approved by the local Spanish institutional review boards and U.S. National Cancer Institute.

*Quantification of LINE-1 methylation*. We extracted granulocyte DNA using standard methods (García-Closas M et al. 2005). We carried out bisulfite conversion of DNA using the EZ-96 DNA Methylation-Gold™ kit (Zymo Research, Irvine, CA, USA) according to the manufacturer’s recommendations. We carried out polymerase chain reaction (PCR) amplification of bisulfite-modified DNA using a set of forward and reverse primers reported previously (Estécio et al. 2007). To quantify the methylation level of each of the first four CpG sites next to the pyrosequencing primer, we performed sequencing of the PCR product by pyrosequencing, using the PyroMark™ Q24 System (QIAGEN, Valencia, CA, USA) as recommended by the manufacturer. The first four were the CpGs from which we could obtain methylation values of all samples. We extracted the methylation level at each CpG site using the PyroMark™ application software, version 2.0.6 (QIAGEN), and we expressed the value as the percentage of methylated cytosines over the sum of methylated and unmethylated cytosines. We used the average methylation level of the first four LINE-1 CpG sites as a surrogate marker of the global DNA methylation level. To determine whether changes in blood cell populations affect LINE-1 methylation levels, we analyzed LINE-1 methylation in independently purified granulocyte and lymphocyte samples. The results showed no significant difference in LINE-1 methylation between granulocyte and lymphocyte samples, thus suggesting that variation in the distribution of peripheral blood cell populations among participants would not contribute to variation in global DNA methylation (data not shown). For the present study, we used DNA extracted from granulocytes to quantify DNA methylation. As a quality control measure, we measured LINE-1 methylation in 129 randomly selected duplicate samples and the within-sample coefficient of variation (CV) was 4.0%. In the analysis, we used the average of the duplicates for those samples.

*Nutritional assessment*. We estimated the usual intake of vitamins B1, B2, B3, B6, and B12, folate, protein, alcohol, fruit, and vegetables over the 5 years before interview using a validated food frequency questionnaire of 127 items (García-Closas R et al. 2007). Micronutrients and macronutrients included in the present analysis have been suggested as important cofactors and methyl donors in one-carbon metabolism (Stover 2009). We calculated nutrient density variables by dividing the total estimated mass of daily food consumed by the total estimated daily energy intake (micrograms per day per kilocalorie).

*Trace elements*. We collected toenail clippings to estimate chronic exposure to trace elements. Sample collection and experimental methods used to measure trace elements level have been reported previously (Amaral et al. 2012). Briefly, after cleaning and washing the toenails to remove external contaminants, we quantified elements at the Trace Element Analysis Core (Dartmouth College, Hanover, NH, USA), using inductively coupled plasma–mass spectrometry (Hopkins et al. 2004). We digested the samples with Optima® Nitric Acid (Fisher Scientific, St. Louis, MO, USA) at 105°C followed by addition of hydrogen peroxide and further heating the dilution with deionized water. We recorded gravimetrically all sample preparation steps. As a quality control, each batch of analyses included six standard reference material samples with known trace element content (GBW 07601, powdered human hair; China National Analysis Center for Iron and Steel, Beijing, China) and six analytic blanks, along with the study samples. In total, we determined concentrations (micrograms per gram) of 12 trace elements (aluminum, arsenic, cadmium, chromium, copper, iron, lead, manganese, nickel, selenium, vanadium, and zinc).

*Genotyping*. For genotype assays, we extracted DNA from leukocytes as described previously (García-Closas M et al. 2005). We determined genotypes at the Core Genotyping Facility of the Division of Cancer Epidemiology and Genetics, National Cancer Institute, MD, USA. We selected for the analysis a total of 515 SNPs in 24 genes involved in the one-carbon metabolism pathway, including DNA methylation and arsenic metabolism [for a list of the 24 genes evaluated, see Supplemental Material, Table S1 (http://dx.doi.org/10.1289/ehp.1206068)]. We selected these genes because they are critical for the one-carbon metabolism pathway (Lee YL et al. 2009; Ulrey et al. 2005). Previously, we described in detail methods of the genotyping process (García-Closas M et al. 2005, 2007; Rothman et al. 2010). We genotyped SNPs using Illumina Infinium® Human1M-Duo, Illumina GoldenGate® (Illumina, San Diego, CA, USA) and TaqMan® (Applied Biosystems, Foster City, CA, USA) assays (for a complete list of SNPs according to assay, see Supplemental Material, Table S2). In addition, we estimated associations between LINE-1 methylation and glutathione *S*-transferase mu 1 (*GSTM1*), glutathione *S*-transferase theta 1 (*GSTT1*), and *N*-acetyltransferase 2 (*NAT2*) variants because of their relevance to bladder cancer (Cash et al. 2012). These variants were determined as described by García-Closas M et al. (2005). All genotypes included in the study were in Hardy-Weinberg equilibrium in the study population (*p* > 0.05) (data not shown).

*Statistical analysis*. The distribution of LINE-1 methylation levels was slightly bimodal and positively skewed [see Supplemental Material, Figure S1 (http://dx.doi.org/10.1289/ehp.1206068)]. To estimate associations between LINE-1 methylation levels and each of the variables considered, we fitted bivariate robust linear regression models and calculated the corresponding beta coefficients and 95% confidence intervals (CIs). Characteristics analyzed as continuous variables were age, micronutrient intakes, fruit and vegetable intakes, and toenail concentrations of trace elements. Characteristics analyzed as categorical variables were BMI (< 25.0, 25.0–26.99, 27.0–29.99, ≥ 30.0), smoking status (non-, occasional, former, current smoker), and tobacco type (nonsmoker, blond only, black only, blond and black, unknown).

To identify SNPs for detailed assessment, we first used the Fisher’s exact test to screen SNPs that were significantly associated (*p* < 0.05) with LINE-1 methylation categorized according to tertiles (< 56.7%, 56.7–58.6%, and > 58.6%) according to codominant mode of inheritance. The 22 SNPs identified for further analyses [listed in Supplemental Material, Table S3 (http://dx.doi.org/10.1289/ehp.1206068)] were subsequently modeled according to all modes of inheritance (additive, codominant, dominant, and recessive). The mode of inheritance that best predicted LINE-1 methylation is reported.

In addition to age and sex, adjusted robust linear regression models for each potential predictor included region, which may be related to diet, micronutrients (Gabriel et al. 2006), and environmental pollution and smoking status, which may be related to trace elements (Moerman and Potts 2011). We also did a sensitivity analysis without adjusting for smoking status to see whether there was a change in the beta estimates. We included all the 515 SNPs in the analysis regardless of linkage disequilibrium. The association between LINE-1 methylation and potential predictors was assessed in the multivariable adjusted model stratifying by sex. Because arsenic and rs7085104 in arsenic (+3 oxidation state) methyltransferase (*AS3MT*), which is involved in arsenic metabolism, were individually associated with LINE-1 methylation, we assessed the presence of effect modification by including a multiplicative interaction term in a model adjusted for age, sex, region, and smoking status. The Wald test was used to calculate the interaction *p*-value. We corrected for multiple testing using the Bonferroni method. We conducted a sensitivity analysis, excluding 42 individual participants who had a CV > 4% for LINE-1 methylation in duplicate samples. This exclusion did not result in substantial differences in the beta coefficients; therefore, those participants remained in the analyses (data not shown). All statistical tests were two-sided, and *p* ≤ 0.05 was considered significant. We carried out all data analyses using Stata/SE version 10.1 (StataCorp, College Station, TX, USA).

## Results

The characteristics of the 892 participants in the present study and median and mean LINE-1 methylation levels according to each variable of interest are provided in Supplemental Material, Table S4 (http://dx.doi.org/10.1289/ehp.1206068). The majority of the study participants were male (89%) and regular (former or current) smokers (63.9%), with a median age of 66 years [interquartile range (IQR) = 13]. The mean LINE-1 methylation level was 58.9% (SD = 5.3%) with minimum and maximum value of 37.9% and 85.7%, respectively. Table 1 shows the association between LINE-1 methylation levels and characteristics of study subjects. In the bivariable robust linear regression analysis, only toenail arsenic and nickel concentrations were significantly associated with LINE-1 methylation. However, in multivariable robust linear regression models adjusted for age, sex, region, and smoking status, the levels of LINE-1 methylation were significantly lower in women than in men (adjusted β = –0.7; 95% CI: –1.2, –0.1, *p* = 0.02) and in smokers of blond tobacco only (adjusted β = –0.7; 95% CI: –1.3, –0.08, *p* = 0.03) and of both blond and black tobacco (adjusted β = –0.6; 95% CI: –1.1, –0.07, *p* = 0.03) compared with nonsmokers. Toenail arsenic concentration also was negatively associated with LINE-1 methylation (adjusted β for a 1-µg/g increase = –3.6; 95% CI: –5.9, –1.2, *p* = 0.003). In contrast, LINE-1 levels were positively associated with 1-µg/g increases in toenail concentrations of iron (adjusted β = 0.002; 95% CI: 0.001, 0.004, *p* = 0.009) and nickel (adjusted β = 0.02; 95% CI: 0.005, 0.03, *p* = 0.004). BMI, B vitamins, folate, total protein, alcohol, and fruit and/or vegetable intake were not significantly associated with LINE-1 methylation regardless of adjustment for covariates (Table 1). Results from the multivariable analyses without adjusting for smoking status done as a sensitivity analysis were not different from the associations above (see Supplemental Material, Table S5). If we were to correct for multiple comparisons by Bonferroni method, none of the above would be significant.

**Table 1:** Association between LINE-1 methylation level and individual characteristics of study subjects in the SBC/EPICURO study.

Variables	*n*	LINE-1 methyla­tion mean (95% CI)	Unadjusted		Adjusted		
			β (95% CI)	*p*-Value	*n*	β (95% CI)^*a,b*^	*p*-Value
Age (years)	892	—	–0.004 (–0.02, 0.01)	0.6	889	–0.006 (–0.02, 0.01)	0.5
Sex
Men	792	59.0 (58.6, 59.4)	Ref		789	Ref
Women	100	58.0 (57.1, 58.8)	–0.4 (–0.9, 0.05)	0.08	100	–0.7 (–1.2, –0.1)	0.02
Region
Barcelona	168	59.3 (58.5, 60.2)	Ref		168	Ref
Vallès	135	58.3 (57.5, 59.0)	–0.2 (–0.7, 0.4)	0.5	135	–0.2 (–0.7, 0.3)	0.5
Elche	73	58.2 (57.1, 59.3)	–0.4 (–1.1, 0.2)	0.2	73	–0.5 (–1.1, 0.2)	0.1
Tenerife	145	58.6 (57.8, 59.4)	–0.1 (–0.7, 0.4)	0.7	144	–0.1 (–0.6, 0.4)	0.7
Asturias	371	59.2 (58.6, 59.8)	0.2 (–0.3, 0.6)	0.5	369	0.1 (–0.3, 0.6)	0.6
BMI (kg/m²)
< 25.0	372	58.8 (58.3, 59.3)	Ref		370	Ref
25.0–26.99	148	58.9 (57.9, 59.9)	–0.2 (–0.6, 0.3)	0.5	148	–0.2 (–0.6, 0.3)	0.4
27.0–29.99	120	59.0 (58.1, 59.8)	0.1 (–0.3, 0.6)	0.6	120	0.2 (–0.3, 0.7)	0.4
≥ 30.0	57	58.8 (57.5, 60.2)	–0.08 (–0.7, 0.6)	0.8	57	–0.03 (–0.7, 0.6)	0.9
Missing data	195
Smoking status
Nonsmoker	255	58.2 (57.7, 58.7)	Ref		255	Ref
Occasional smoker	66	60.0 (58.4, 61.5)	0.4 (–0.2, 1.1)	0.2	66	0.3 (–0.3, 1.0)	0.3
Former smoker	329	58.9 (58.3, 59.5)	–0.2 (–0.5, 0.3)	0.6	329	–0.3 (–0.7, 0.1)	0.2
Current smoker	239	59.4 (58.6, 60.2)	–0.2 (–0.6, 0.2)	0.4	239	–0.4 (–0.9, 0.07)	0.1
Missing data
Tobacco type
Nonsmoker	255	58.2 (57.7, 58.7)	Ref		255	Ref
Blond only	99	58.5 (57.6, 59.5)	–0.5 (–1.0, 0.09)	0.1	99	–0.7 (–1.3, –0.08)	0.03
Black only	219	59.6 (58.8, 60.4)	0.06 (–0.4, 0.5)	0.8	218	–0.2 (–0.6, 0.3)	0.5
Blond and black	154	58.7 (57.8, 59.6)	–0.3 (–0.8, 0.2)	0.2	154	–0.6 (–1.1, –0.07)	0.03
Unknown	97	59.3 (58.2, 60.5)	–0.01 (–0.6, 0.6)	0.9	97	–0.12 (–0.7, 0.5)	0.7
Missing data	68
Controls’ diagnosis
Hernia	332	58.8 (58.2, 59.4)	Ref		330	Ref
Fracture and trauma	263	59.3 (58.6, 60.0)	–0.2 (–0.5, 0.2)	0.4	262	–0.02 (–0.4, 0.4)	0.9
Hydrocele	122	58.7 (57.8, 59.6)	0.2 (–0.3, 0.7)	0.4	122	0.2 (–0.3, 0.7)	0.5
Other abdominal surgery	99	58.3 (57.4, 59.2)	–0.2 (–0.7, 0.3)	0.4	99	–0.09 (–0.6, 0.5)	0.7
Other diseases	76	59.2 (57.9, 60.5)	0.01 (–0.9, 0.6)	0.9	76	0.2 (–0.4, 0.8)	0.5
Dietary intake^*c*^
Vitamin B1 (µg/day/kcal)	645	—	0.5 (–0.6, 1.6)	0.3	644	0.6 (–0.6, 1.7)	0.3
Vitamin B2 (µg/day/kcal)	645	—	0.1 (–0.5, 0.8)	0.7	644	0.2 (–0.5, 0.8)	0.6
Vitamin B3 (µg/day/kcal)	645	—	0.01 (–0.05, 0.08)	0.7	644	0.02 (–0.05, 0.09)	0.5
Vitamin B6 (µg/day/kcal)	645	—	0.6 (–0.2, 1.4)	0.1	644	0.8 (–0.05, 1.6)	0.07
Vitamin B12 (µg/day/kcal)	645	—	–0.04 (–0.09, 0.01)	0.1	644	–0.03 (–0.08, 0.02)	0.3
Folate (µg/day/kcal)	645	—	0.001 (–0.002, 0.004)	0.4	644	0.003 (–0.001, 0.01)	0.1
Protein (µg/day/kcal)	645	—	0.01 (–0.01, 0.03)	0.4	644	0.01 (–0.01, 0.03)	0.4
Alcohol (µg/day/kcal)	645	—	–0.002 (–0.02, 0.02)	0.8	644	–0.01 (–0.03, 0.02)	0.5
Fruit (g/day/kcal)	639	—	0.0001 (–0.001, 0.002)	0.9	638	0.0001 (–0.001, 0.002)	0.9
Vegetable (g/day/kcal)	640	—	0.001 (–0.001, 0.003)	0.3	639	0.002 (–0.001, 0.004)	0.1
Fruit and vegetable (g/day/kcal)	639	—	0.0003 (–0.0008, 0.001)	0.6	638	0.0005 (–0.0007, 0.002)	0.4
Toenail trace elements (µg/g)^*d*^
Aluminum	658	—	–0.003 (–0.008, 0.002)	0.2	658	–0.003 (–0.008, 0.002)	0.2
Arsenic	659	—	–2.9 (–5.2, –0.6)	0.02	659	–3.6 (–5.9, –1.2)	0.003
Cadmium	659	—	0.08 (–0.4, 0.5)	0.7	659	0.1 (–0.3, 0.6)	0.6
Chromium	658	—	0.06 (–0.01, 0.1)	0.09	659	–0.01 (–0.05, 0.03)	0.6
Copper	659	—	–0.002 (–0.06, 0.05)	0.95	659	–0.01 (–0.07, 0.04)	0.6
Iron	657	—	–0.002 (–0.006, 0.002)	0.4	658	0.002 (0.001, 0.004)	0.009
Lead	659	—	–0.05 (–0.1, 0.03)	0.2	659	–0.06 (–0.1, 0.02)	0.2
Manganese	659	—	–0.03 (–0.1, 0.09)	0.7	659	–0.05 (–0.2, 0.06)	0.4
Nickel	659	—	0.02 (0.006, 0.03)	0.002	659	0.02 (0.005, 0.03)	0.004
Selenium	659	—	0.1 (–0.8, 1.0)	0.8	659	0.2 (–0.7 1.2)	0.6
Vanadium	651	—	–0.7 (–2.7, 1.3)	0.5	651	–0.9 (–2.8, 1.2)	0.4
Zinc	659	—	–0.002 (–0.004, 0.001)	0.2	659	–0.001 (–0.004, 0.002)	0.4
*NAT2* phenotype
Rapid/intermediate acetylator	389	59.0 (58.4, 59.6)	Ref		388	Ref
Slow acetylator	498	58.8 (58.4, 59.2)	0.2 (–0.1, 0.5)	0.3	496	0.2 (–0.1, 0.5)	0.2
Missing data
*GSTM1* genotype
(+/+, +/–)	421	58.9 (58.3, 59.4)	Ref		419	Ref	
(–/–)	462	59.0 (58.5, 59.4)	0.04 (–0.3, 0.4)	0.8	461	0.01 (–0.3, 0.3)	0.9
Missing data	9						
*GSTT1* genotype
(+/+, +/–)	688	59.0 (58.6, 59.4)	Ref		685	Ref	
(–/–)	198	58.5 (57.9, 59.2)	–0.2 (–0.6, 0.2)	0.3	198	–0.2 (–0.6, 0.2)	0.4
Missing data	6						
The exposure contrast for trace elements is 1 μg/g and for dietary variables is 1 μg/day/kcal. ^***a***^Adjusted for age, sex, region, and smoking status (non-, occasional, former, current smoker). Tobacco type’s β is not adjusted for smoking status.^***b***^The number of observations are reduced by three because of missing data on smoking status.^***c***^Data available for those who completed food frequency questionnaire.^***d***^Data available for those who provided toenail for trace element assessment.							

Out of the 515 genetic variants assessed, 22 passed a first screening using Fisher’s exact test (*p* ≤ 0.05 according to codominant mode of inheritance) [see Supplemental Material, Table S3 (http://dx.doi.org/10.1289/ehp.1206068)]. Of these, 7 SNPs in five genes were significantly associated with LINE-1 methylation based on multivariable models adjusted for age, sex, region, and smoking status (Table 2; model-based estimates for the 15 SNPs that were not significantly associated with LINE-1 methylation are reported in Supplemental Material, Table S6.) Significant positive associations were estimated for DNA (cytosine-5-)-methyltransferase 3 alpha (*DNMT3A*)*-*rs7581217 (per allele: adjusted β = 0.3; 95% CI: 0.1, 0.6, *p* = 0.002); transcobalamin II (*TCN2*)*-*rs9621049 (recessive: adjusted β = 4.2; 95% CI: 2.8, 5.7, *p* = 4.7 × 10^–9^), *TCN2-*rs4820887 (recessive: adjusted β = 4.0; 95% CI: 2.5, 5.6, *p* = 4.8 × 10^–7^) and *TCN2-*rs9606756 (recessive: adjusted β = 1.9; 95% CI: 0.5, 3.3, *p* = 0.008)*;* and *AS3MT*-rs7085104 (recessive: adjusted β = 0.7; 95% CI: 0.3, 1.2, *p* = 0.001). In addition, significant associations under the codominant mode of inheritance (based on global *p*-values) were estimated for solute carrier family 19 (folate transporter), member 1 (*SLC19A1*)*-*rs914238 (TC vs. TT, β = 0.5; 95% CI: 0.08, 0.8; CC vs. TT, β = –0.3; 95% CI: –0.7, 0.2; global *p* = 0.0007) and 5,10-methenyltetrahydrofolate synthetase (*MTHFS*)-rs1380642 (CT vs. CC, β = 0.3; 95% CI: –0.08, 0.6; TT vs. CC, β = –0.8; 95% CI: –1.6, 0.09; global *p* = 0.05). After correcting for multiple testing using the Bonferroni method, *TCN2-*rs9621049 and *TCN2-*rs4820887 remained significant (*p* < 0.05).

**Table 2:** Association between LINE-1 methylation levels and SNPs in genes involved in the one-carbon metabolism pathway.

Gene	dbSNP [chromosome, position in the gene, location^*a*^]	MAF	*n*	MOI	Genotype	Unadjusted		Adjusted	
						β (95% CI)	*p*-Value	β^*b*^ (95% CI)	*p*-Value
*DNMT3A*	rs7581217 [2, intron, 25378448]	0.39	875	Additive	per allele T	0.3 (0.1, 0.6)	0.003	0.3 (0.1, 0.6)	0.002
*AS3MT*	rs7085104 [10, flanking 5’UTR, 104618863]	0.38	751	Recessive	AA/AG	Ref		Ref
			124		GG	0.8 (0.3, 1.2)	0.0008	0.7 (0.3, 1.2)	0.001
*MTHFS*^*c*^	rs1380642 [15, flanking 3’UTR, 77883926]	0.18	585	Codominant	CC	Ref	0.03	Ref	0.05
			258		CT	0.3 (–0.05, 0.6)		0.3 (–0.08, 0.6)	
			32		TT	–0.8 (–1.6, 0.07)		–0.8 (–1.6, 0.09)	
*SLC19A1*^*c*^	rs914238 [21, flanking 5’UTR, 45840089]	0.49	231	Codominant	TT	Ref	0.0008	Ref	0.0007
			435		TC	0.5 (0.09, 0.8)		0.5 (0.08, 0.8)	
			209		CC	–0.2 (–0.7, 0.2)		–0.3 (–0.7, 0.2)	
*TCN2*^*d*^	rs9621049 [22, exon, 29343419]	0.11	864	Recessive	CC/CT	Ref		Ref
			11		TT	4.5 (3.1, 5.9)	4.3 × 10^–10^	4.2 (2.8, 5.7)	4.7 × 10^–9^
	rs9606756 [22, exon, 29336860]	0.12	864	Recessive	AA/AG	Ref		Ref
			11		GG	2.2 (0.8, 3.6)	0.003	1.9 (0.5, 3.3)	0.008
	rs4820887 [22, intron, 29346914]	0.10	866	Recessive	GG/GA	Ref		Ref
			9		AA	4.6 (3.0, 6.2)	9.3 × 10^–9^	4.0 (2.5, 5.6)	4.8 × 10^–7^
Abbreviations: MAF, minor allele frequency; MOI, mode of inheritance. ^***a***^Human Genome Build 36.3 location.^***b***^Adjusted for age, sex, region, and smoking status.^***c***^Global*p*-value for rs1380642 and rs914238 was estimated by using a two-degree-of-freedom likelihood-ratio test.^***d***^Linkage disequilibrium (rs4820887 vs. rs9621049)*r*^2^ = 0.8.									

A significant interaction (*p* = 0.01) was observed between arsenic and *AS3MT*-rs7085104 on LINE-1 methylation (adjusted β for a 1-μg/g increase in As = –4.1; 95% CI: –6.6, –1.7, *p* = 0.001 for genotype AA/AG; and adjusted β for a 1-μg/g increase in As = 10.2; 95% CI: –3.2, 23.7, *p* = 0.1 for genotype GG).

After simultaneously adjusting for age, geographic region, and all factors that were significant predictors of LINE-1 methylation (sex; tobacco type; toenail arsenic, iron, and nickel; and the 5 SNPs noted above), associations with sex, arsenic, nickel, iron, *DNMT3A-*rs7581217, *TCN2-*rs9621049, and *MTHFS*-rs1380642 remained significant. The association with blond tobacco was nonsignificant although the direction of the point estimate remained unchanged. The association between rs9606756, rs4820887, and LINE-1 methylation become nonsignifcant [see Supplemental Material, Table S7 (http://dx.doi.org/10.1289/ehp.1206068)]. This might be due to reduced sample size in the simultaneously adjusted model due to missing data.

## Discussion

In the present study, we used a comprehensive approach to assess associations of genetic and non-genetic factors with LINE-1 methylation in a group of participants 20–81 years of age. Lower levels of LINE-1 methylation were found among women compared with men, and among smokers of blond tobacco compared with nonsmokers. In addition, toenail concentrations of arsenic were also negatively associated with LINE-1 methylation. On the other hand, LINE-1 methylation levels were positively associated with toenail concentrations of iron and nickel, and with seven variants in *DNMT3A, TCN2, AS3MT, SLC19A1,* and *MTHFS* genes.

Our findings support previous results showing that women have significantly lower levels of LINE-1 methylation (El-Maarri et al. 2007, 2011; Wilhelm et al. 2010; Zhu et al. 2012). DNA methylation is important for X-chromosome inactivation in women (Jones and Liang 2009), and although LINE-1 sequences do not seem to be the major mechanism involved in this process, they may be involved in spreading the X-inactivation signal across the chromosome (Bailey et al. 2000). In support of this, a recent study showed that LINE-1 sequences were hypomethylated in the inactive X-chromosome (Singer et al. 2012). A small study of 33 men and 33 women reported lower levels of blood SAM in women (Poirier et al. 2001). Hormonal factors may also contribute to the difference in methylation levels between sexes. However, a recent *in vitro* study assessing the roles of estrogen, progesterone, and dihydrotestosterone on DNA methylation in four cell lines found no detectable effect of these hormones on methylation levels at the LINE-1 and *Alu* repeats (El-Maarri et al. 2011). Further studies are needed to decipher the relationship between sex and LINE-1 methylation.

Because tobacco smoking is an important contributor to disease and is a modifiable behavioral factor, there has been much interest in the relationship between smoking and DNA methylation. Our findings are in line with other studies that reported no association between LINE-1 methylation and smoking status (Terry et al. 2011). In the present study, we found that subjects who smoked blond tobacco had lower levels of global DNA methylation than nonsmokers. An experimental study has shown that cigarette smoke condensates can induce DNA demethylation in repeat elements such as LINE-1 and D4Z4 (Liu et al. 2010). Both black and blond tobacco cause disease although the former is more mutagenic, reflective of the higher levels of *N*-nitrosamines and aromatic amines in smoke produced by black tobacco (Malaveille et al. 1989). Our findings suggest that the toxic effects of blond tobacco could be mediated by modulating the epigenetic landscape. This may have a public health implication given epigenetic alterations are reversible.

We also provide evidence that arsenic levels were inversely associated with LINE-1 methylation, and that arsenic may have a strong effect on LINE-1 methylation. For every 1-μg/g increase in arsenic there was a 3.6% decrease in DNA methylation level. This inverse association is in agreement with that from a population-based study that used a similar assay to assess LINE-1 methylation levels and toenail concentrations of arsenic (Wilhelm et al. 2010), as well as with several other experimental studies (Reichard and Puga 2010; Ren et al. 2011). The mechanisms through which arsenic exposure influences DNA methylation are not fully understood. Studies in cell lines and mouse models exposed to arsenic for ≤ 22 and 48 weeks, respectively, have shown that prolonged exposure to sodium arsenite resulted in decreased global DNA methylation, and inhibition of DNA (cytosine-5-)-methyltransferase 1 (DNMT1), DNMT3A, and DNA (cytosine-5-)-methyltransferase 3 beta (DNMT3B) gene expression (Reichard and Puga 2010; Ren et al. 2011). It is likely that through the combined effect of depleting the cellular pool of SAM and inhibiting the activity of DNMTs, both inorganic and organic arsenic may decrease global DNA methylation.

We are not aware of any human studies associating iron and nickel levels and global DNA methylation. In the present study, iron and nickel showed a small but significant positive association with LINE-1 methylation level. Genes involved in hepatocellular carcinoma (HCC) have been found to be hypermethylated in hereditary hemochromatosis, a disease characterized by chronic iron overload that is a risk factor for HCC (Lehmann et al. 2007). Iron, together with 2-oxoglutarate and oxygen, is an essential cofactor for the ten-eleven translocation (TET) family of proteins that hydroxylate 5-methylcytosine to 5-hydroxymethylcytosine and further oxidize to 5-carboxylcytosine and 5-formylcytosine, which have all been suggested to be precursors for both active and passive DNA demethylation (Bhutani et al. 2011). Experimental studies conducted in Chinese hamster cell lines (G12) treated with nickel chloride for up to 3 weeks have shown that nickel chloride treatment leads to both promoter hypermethylation and elevated total genomic DNA methylation (Lee YW et al. 1995, 1998). How nickel induces DNA methylation is not yet understood, but it has been proposed that nickel first induces chromatin condensation followed by *de novo* methylation of heterochromatic DNA (Lee YW et al. 1995).

The three SNPs with the strongest associations with LINE-1 methylation were all in *TCN2*, including two exonic SNPs that result in missense substitutions (rs9606756 and rs9621049) and one intronic SNP (rs4820887). SNPs rs9621049 and rs4820887 have a linkage disequilibrium *r*^2^ value of 0.8, implying that the observed effect in LINE-1 methylation may eventually be attributed to either of them. *TCN2* encodes for transcobalamin II which binds and transports vitamin B12 into the cell (Regec et al. 1995), suggesting that variations in *TCN2* could potentially impair the one-carbon metabolism pathway by altering the cytoplasmic concentration of vitamin B12. *TCN2*-rs9606756 leads to an I23V substitution located at a NAGNAG tandem acceptor site that is a target of alternative splicing (Hiller et al. 2006). *TCN2*-rs9621049 leads to a S348F and may also play a role in the availability of vitamin B12 in the cell thereby affecting LINE-1 methylation levels.

Four SNPs in other genes (*DNMT3A*-​rs7581217, *AS3MT*-rs7085104, *MTHFS*-​rs1380642, *SLC19A1*-rs914238) involved in the one-carbon metabolism were also associated with global DNA methylation in our study population. DNMT3A, a *de novo* DNA methyltransferase, establishes the patterns of methylation in early embryonic development, along with DNMT3B, and cooperates with DNMT1 to maintain the methylation of repetitive sequences such as LINE-1 and *Alu* elements (Jones and Liang 2009). Recurrent mutations in *DNMT3A* have been associated with adult hematologic malignancies (Ley et al. 2010; Yan et al. 2011), and mice lacking *Dnmt3a* die within the first weeks of postnatal life (Robertson 2005). The product of *AS3MT* catalyzes the conversion of trivalent arsenic by adding a methyl group to monomethylarsonic acid and dimethylarsonic acid (Ren et al. 2011); monomethylarsonic acid being the most toxic metabolite (Engström et al. 2011). rs7085104, located in the promoter region of *AS3MT*, has been associated with arsenic metabolism, as evidenced by differences in urinary concentration of arsenic metabolites (Engström et al. 2011; Valenzuela et al. 2009). We also observed a significant interaction of this SNP with levels of arsenic on LINE-1 methylation levels. Whereas subjects with at least one copy of the major allele had a 4.1% decrease in methylation level per 1-μg/g increase in arsenic, which is comparable to the overall population (–3.6%), those homozygous for the variant allele had a 10% increase in LINE-1 methylation. These findings support the putative functionality of the association. The product of *MTHFS* catalyzes the conversion of 5-formyltetrahydrofolate to 5,10-methenyltetrahydrofolate and a genome-wide association study reported an association between a variant in this gene and chronic kidney disease (Kottgen et al. 2008). *SLC19A1* is a ubiquitously expressed major transporter of folate and antifolates and regulator of the intracellular concentrations of folate (Matherly et al. 2007). Common variants in this gene have been associated with plasma folate levels, various types of cancer (esophageal, gastric, and acute lymphoblastic leukemia), and altered methotrexate transport and adverse effects of methotrexate (Matherly et al. 2007).

Among the limitations of the study is that the majority of paraticipants were of advanced age (mean = 64 years of age, SD = 10 years) and male. This may explain the lack of association between DNA methylation and age in our study population, in contrast with other studies that included subjects with a broader age range (Fraga et al. 2005). Thus, our findings refer to an adult population of mostly men. Results were consistent with estimates for the population as a whole when stratified by sex, with the exception of nickel, tobacco type, and *MTHFS*-rs1380642, which become nonsignificant when the point estimates were in the same direction (data not shown). These differences may reflect reduced power to estimate associations among women as a result of the small sample size. The presence of missing data for some of the variables might have resulted in decreased power, but even with the available sample size we were able to reproduce previous results and identify novel predictors of LINE-1 methylation. Furthermore, although the study subjects were recruited from hospitals, none of reasons for hospitalization were significantly associated with LINE-1 methylation.

Strengths of the study include its size and the availability and quality of individual data on demographics, lifestyle, environmental exposures, and genetics. Additionally, we assessed LINE-1 methylation levels, which are considered a good marker of global DNA methylation (Yang et al. 2004), using pyrosequencing, which gives accurate and reproducible measurements (Estécio et al. 2007; Laird 2010; Tost and Gut 2007). Furthermore, this assessment was made using DNA from granulocytes, thereby avoiding a possible effect of cell blood count in our study.

To the best of our knowledge, this is the first study to identify seven SNPs in association with changes in LINE-1 methylation and to integrate different types of information to assess the determinants of global methylation in blood DNA. Integration of both internal and external exposure data in this study is a step forward in understanding how the exposome modulates DNA methylation patterns.

## Conclusions

The present study provides further evidence that DNA methylation levels are influenced by variants in genes involved in the one-carbon metabolism pathway, and exposure to trace elements and tobacco smoke. Given the fact that smoking and some of the genetic variants and trace elements associated with LINE-1 methylation in the present study have also been associated with adverse health outcomes including cancer, our results provide additional insight into the potential mechanism through which these agents participate in the development of those diseases. Furthermore, these factors should be considered as potential confounders in etiologic and interventional studies analyzing the role of DNA methylation in disease. Nevertheless, future studies are required to replicate and extend our findings in different populations.

## Appendix 1

**Spanish Bladder Cancer/EPICURO Study investigators**

Institut Municipal d’Investigació Mèdica, Universitat Pompeu Fabra, Barcelona–Coordinating Center (M. Kogevinas, N. Malats, F.X. Real, M. Sala, G. Castaño, M. Torà, D. Puente, C. Villanueva, C. Murta-Nascimento, J. Fortuny, E. López, S. Hernández, R. Jaramillo, G. Vellalta, L. Palencia, F. Fermández, A. Amorós, A. Alfaro, G. Carretero); Hospital del Mar, Universitat Autònoma de Barcelona, Barcelona (J. Lloreta, S. Serrano, L. Ferrer, A. Gelabert, J. Carles, O. Bielsa, K. Villadiego); Hospital Germans Trias i Pujol, Badalona, Barcelona (L. Cecchini, J.M. Saladié, L. Ibarz); Hospital de Sant Boi, Sant Boi de Llobregat, Barcelona (M. Céspedes); Consorci Hospitalari Parc Taulí, Sabadell (C. Serra, D. García, J. Pujadas, R. Hernando, A. Cabezuelo, C. Abad, A. Prera, J. Prat); Centre Hospitalari i Cardiològic, Manresa, Barcelona (M. Domènech, J. Badal, J. Malet); Hospital Universitario de Canarias, La Laguna, Tenerife (R. García-Closas, J. Rodríguez de Vera, A.I. Martín); Hospital Universitario Nuestra Señora de la Candelaria, Tenerife (J. Taño, F. Cáceres); Hospital General Universitario de Elche, Universidad Miguel Hernández, Elche, Alicante (A. Carrato, F. García-López, M. Ull, A. Teruel, E. Andrada, A. Bustos, A. Castillejo, J.L. Soto); Universidad de Oviedo, Oviedo, Asturias (A. Tardón); Hospital San Agustín, Avilés, Asturias (J.L. Guate, J.M. Lanzas, J. Velasco); Hospital Central Covadonga, Oviedo, Asturias (J.M. Fernández, J.J. Rodríguez, A. Herrero); Hospital Central General, Oviedo, Asturias (R. Abascal, C. Manzano, T. Miralles); Hospital de Cabueñes, Gijón, Asturias (M. Rivas, M. Arguelles); Hospital de Jove, Gijón, Asturias (M. Díaz, J. Sánchez, O. González); Hospital de Cruz Roja, Gijón, Asturias (A. Mateos, V. Frade); Hospital Alvarez-Buylla, Mieres, Asturias (P. Muntañola, C. Pravia); Hospital Jarrio, Coaña, Asturias (A.M. Huescar, F. Huergo); Hospital Carmen y Severo Ochoa, Cangas, Asturias (J. Mosquera).

## Supplemental Material

(328 KB) PDF
