# Supplementary material for: Genetic and Non-genetic Predictors of LINE-1 Methylation in Leukocyte DNA
Source: Environ Health Perspect. 2013 Apr 3;121(6):650–6. doi: 10.1289/ehp.1206068 (PMC3672919; doi:10.1289/ehp.1206068)
Supplement: (328 KB) PDF [file ehp.1206068.s001.pdf]

# Supplemental Material

# Genetic and Non-genetic Predictors of LINE-1 Methylation in Leukocyte DNA

Salman M. Tajuddin[1], André F. S. Amaral[1], Agustín F. Fernández[2], Sandra Rodríguez-Rodero[2], Ramón María Rodríguez[2], Lee E. Moore[3], Adonina Tardón[4], Alfredo Carrato[5,6], Montserrat García-Closas[7], Debra T. Silverman[3], Brian P. Jackson[8], Reina García-Closas[9], Ashley L. Cook[1], Kenneth P. Cantor[10], Stephen Chanock[3], Manolis Kogevinas[11], Nathaniel Rothman[3], Francisco X. Real[12,13], Mario F. Fraga[2,14*], Núria Malats[1*] for the Spanish Bladder Cancer (SBC)/EPICURO Study investigators[15]

[1]Genetic and Molecular Epidemiology Group, Spanish National Cancer Research Centre (CNIO), Madrid, Spain

[2]Cancer Epigenetics Laboratory, Instituto Universitario de Oncología del Principado de Asturias (IUOPA), HUCA, Universidad de Oviedo, Oviedo, Spain

[3]National Cancer Institute, Bethesda, Maryland, USA

[4]Universidad de Oviedo, Oviedo, Spain

[5]Hospital General Universitario de Elche, Elche, Spain

[6]Hospital Ramon y Cajal, Madrid, Spain

[7]Division of Genetics and Epidemiology, Institute of Cancer Research, Sutton, United Kingdom

[8]Trace Element Analysis Core, Department of Earth Sciences, Dartmouth College, Hanover, New Hampshire, USA

[9]Unidad de Investigación, Hospital Universitario de Canarias, La Laguna, Spain

[10]KP Cantor Environmental LLC, Silver Spring, Maryland, USA

[11]Centre for Research in Environmental Epidemiology (CREAL), Barcelona, Spain

[12]Epithelial Carcinogenesis Group, Spanish National Cancer Research Centre (CNIO), Madrid, Spain

[13]Departament de Ciències Experimentals i de la Salut, Universitat Pompeu Fabra, Barcelona, Spain

[14]Department of Immunology and Oncology, Centro Nacional de Biotecnología/CNB-CSIC, Cantoblanco, Madrid, Spain

[15]Spanish Bladder Cancer (SBC)/EPICURO Study investigators are listed in Appendix 1

* Co-last authors contributed equally to this work.

Address Correspondence to:

Núria Malats, M.D., Ph.D.

Genetic and Molecular Epidemiology Group

Spanish National Cancer Research Centre (CNIO)

C/ Melchor Fernández Almagro, 3

28029 Madrid, Spain

Phone: +34-912-246-900 (ext. 3330)

Fax: +34-912-246-911

E-mail: nmalats@cnio.es

Table of Contents Page

Supplemental Material, Table S1…………………………………………………………4

Supplemental Material, Table S2…………………………………………………………7

Supplemental Material, Table S3…………………………………………………………8

Supplemental Material, Table S4…………………………………………………………9

Supplemental Material, Table S5…………………………………………………………13

Supplemental Material, Table S6…………………………………………………………15

Supplemental Material, Table S7…………………………………………………………16

Supplemental Material, Figure S1………………………………………………………..17

**Supplemental Material, Table S1. List of genes involved in the one-carbon metabolism pathway selected for the present study**

| Chromosome location | Gene symbol [ID][a] | Gene name | Gene function |
|---|---|---|---|
| 1p31.1 | *CTH* | Cystathionase | Converts cystathione derived from methionine into cysteine. |
| 1p36.3 | *MTHFR* | Methylenetetrahydrofolate reductase (NAD(P)H) | Catalyzes the conversion of 5,10-methylenetetrahydrofolate to 5-methyltetrahydrofolate. |
| 1q43 | *MTR* | 5-methyltetrahydrofolate-homocysteine methyltransferase | Catalyzes the final step in methionine biosynthesis. |
| 2p23 | *DNMT3A* | DNA (cytosine-5-)-methyltransferase 3 alpha | Functions in de novo methylation of cytosine residues. |
| 2q35 | *ATIC* | 5-aminoimidazole-4-carboxamide ribonucleotide formyltransferase/IMP(inosine monophosphate) cyclohydrolase | Catalyzes the last two steps of the de novo purine biosynthetic pathway. The N-terminal domain has phosphoribosylaminoimidazolecarboxamide formyltransferase activity, and the C-terminal domain has IMP cyclohydrolase activity. |
| 3p21.1 | *CHDH* | Choline dehydrogenase | Encodes a protein that localizes to the mitochondrion. Variations in this gene can affect susceptibility to choline deficiency. |
| 3q21.3 | *ALDH1L1* | Aldehyde dehydrogenase 1 family, member L1 | Catalyzes the conversion of 10-formyltetrahydrofolate, NADP, and water to tetrahydrofolate, NADPH, and carbon dioxide. |
| 5p15.31 | *MTRR* | 5-methyltetrahydrofolate-homocysteine methyltransferase reductase | Regenerates a functional methionine synthase via reductive methylation |
| 5q11.2-q13.2 | *DHFR* | Dihydrofolate reductase | Converts dihydrofolate into tetrahydrofolate, a methyl group shuttle required for the de novo synthesis of purines, thymidylic acid, and certain amino acids. |
| 5q13.1-q15 | *BHMT* | Betaine-homocysteine S-methyltransferase | Catalyzes the conversion of betaine and homocysteine to dimethylglycine and methionine, respectively. |
| 6p12 | *GNMT* | Glycine N-methyltransferase | Catalyzes the conversion of S-adenosyl-L-methionine (along with glycine) to S-adenosyl-L-homocysteine and sarcosine. |

| Chromosome location | Gene symbol [ID][a] | Gene name | Gene function |
|---|---|---|---|
| 10q24.32 | *AS3MT* | Arsenic (+3 oxidation state) methyltransferase | Catalyzes the transfer of a methyl group from S-adenosyl-L-methionine to trivalent arsenical and play a role in arsenic metabolism. |
| 11p11.2 | *FOLH1* | Folate hydrolase (prostate-specific membrane antigen)1/glutamate carboxypeptidase II/ | Acts as a glutamate carboxypeptidase on different alternative substrates, including the nutrient folate and the neuropeptide N-acetyl-l-aspartyl-l-glutamate. |
| 14q24 | *MTHFD* | Methylenetetrahydrofolate dehydrogenase (NADP+ dependent) 1 | Catalyzes one of three sequential reactions in the interconversion of 1-carbon derivatives of tetrahydrofolate. |
| 15q25.1 | *MTHFS* | 5,10-methenyltetrahydrofolate synthetase | Catalyzes the conversion of 5-formyltetrahydrofolate to 5,10-methenyltetrahydrofolate, a precursor of reduced folates involved in 1-carbon metabolism. |
| 17p11.2 | *PEMT* | Phosphatidylethanolamine N-methyltransferase | Converts phosphatidylethanolamine to phosphatidylcholine by sequential methylation in the liver. |
| 17p11.2 | *SHMT1* | Serine hydroxymethyltransferase 1 | Catalyzes the reversible conversion of serine and tetrahydrofolate to glycine and 5,10-methylene tetrahydrofolate. This reaction provides one carbon units for synthesis of methionine, thymidylate, and purines in the cytoplasm. |
| 18p11.32 | *TYMS* | Thymidylate synthetase | Catalyzes the methylation of deoxyuridylate to deoxythymidylate using 5,10-methylenetetrahydrofolate as a cofactor. |
| 19p13.2 | *DNMT1* | DNA (cytosine-5-)-methyltransferase 1 | Establishes and regulates tissue-specific patterns of methylated cytosine residues. |
| 20q11.2 | *DNMT3B* | DNA (cytosine-5-)-methyltransferase 3 beta | Functions in de novo methylation of cytosine residues. |
| 21q22.1; 21q22.11 | *GART* | Glycinamide ribonucleotide transformylase | Encodes a protein that has phosphoribosylglycinamide formyltransferase, phosphoribosylglycinamide synthetase, phosphoribosylaminoimidazole synthetase activity which is required for de novo purine biosynthesis. |
| 21q22.3 | *CBS* | Cystathionine-beta-synthase | Acts as a homotetramer to catalyze the conversion of homocysteine to cystathionine, the first step in the |

| Chromosome location | Gene symbol [ID][a] | Gene name | Gene function |
|---|---|---|---|
| | | | transsulfuration pathway. |
| 21q22.3 | *SLC19A1* | Solute carrier family 19 (folate transporter), member 1 | Transports folate and is involved in the regulation of intracellular concentrations of folate. |
| 22q12.2 | *TCN2* | Transcobalamin II | Binds and mediates the transport of vitamin B12 into cells. |

[a]Maglott D, Ostell J, Pruitt KD, Tatusova T. 2005. Entrez Gene: gene-centered information at NCBI. Nucleic Acids Res 33:D54-D58.

**Supplemental Material, Table S2. Distribution of SNPs studied in the present study by genotyping platform.**

This table is provided as a separate Excel file.

**Supplemental Material, Table S3. List of single nucleotide polymorphisms with significant Fisher's exact test selected for further analysis (p-value ≤ 0.05)**

| dbSNP Identifier | N | MAF | Chromosome | Location[a] | Gene | Region in the gene | P-HWE | Fisher's exact test p-value[b] |
|---|---|---|---|---|---|---|---|---|
| rs11683424 | 875 | 0.12 | 2 | 25342636 | *DNMT3A* | Intron | 0.20 | 0.006 |
| rs7581217 | 875 | 0.39 | 2 | 25378448 | *DNMT3A* | Intron | 0.62 | 0.002 |
| rs1550117 | 875 | 0.08 | 2 | 25419411 | *DNMT3A* | Flanking 5'UTR | 0.81 | 0.009 |
| rs4369857 | 875 | 0.04 | 2 | 215841917 | *ATIC* | Flanking 5'UTR | 1.00 | 0.02 |
| rs7563206 | 874 | 0.47 | 2 | 215898899 | *ATIC* | Intron | 0.84 | 0.04 |
| rs1880586 | 875 | 0.47 | 2 | 215903416 | *ATIC* | Intron | 0.89 | 0.04 |
| rs4673991 | 875 | 0.32 | 2 | 215920334 | *ATIC* | Intron | 0.31 | 0.004 |
| rs4673993 | 875 | 0.32 | 2 | 215920584 | *ATIC* | Intron | 0.31 | 0.004 |
| rs4672768 | 873 | 0.32 | 2 | 215922369 | *ATIC* | Intron | 0.31 | 0.005 |
| rs10498036 | 875 | 0.40 | 2 | 215922737 | *ATIC* | Flanking 3'UTR | 0.57 | 0.004 |
| rs7604984 | 875 | 0.40 | 2 | 215925884 | *ATIC* | Flanking 3'UTR | 0.62 | 0.003 |
| rs7085104 | 875 | 0.38 | 10 | 104618863 | *AS3MT* | Flanking 5'UTR | 1.00 | 0.02 |
| rs1077965 | 875 | 0.41 | 15 | 77852392 | *MTHFS* | Flanking 3'UTR | 0.58 | 0.007 |
| rs11855092 | 875 | 0.24 | 15 | 77877293 | *MTHFS* | Flanking 3'UTR | 0.31 | 0.03 |
| rs1380642 | 875 | 0.18 | 15 | 77883926 | *MTHFS* | Flanking 3'UTR | 0.57 | 0.01 |
| rs4646340 | 875 | 0.37 | 17 | 17434740 | *PEMT* | Intron | 0.51 | 0.04 |
| rs7215833 | 875 | 0.36 | 17 | 17448033 | *PEMT* | Flanking 5'UTR | 0.51 | 0.02 |
| rs914238 | 875 | 0.49 | 21 | 45840089 | *SLC19A1* | Flanking 5'UTR | 0.89 | 0.002 |
| rs2838965 | 873 | 0.42 | 21 | 45846212 | *SLC19A1* | Flanking 5'UTR | 0.33 | 0.02 |
| rs9606756 | 875 | 0.12 | 22 | 29336860 | *TCN2* | Exon | 0.87 | 0.005 |
| rs9621049 | 875 | 0.11 | 22 | 29343419 | *TCN2* | Exon | 0.86 | 0.02 |
| rs4820887 | 875 | 0.10 | 22 | 29346914 | *TCN2* | Intron | 0.85 | 0.01 |

MAF, minor allele frequency. P-HWE, Chi-square test p-value for Hardy-Weinberg equilibrium.

[a]Human Genome Build 36.3 location.

[b]Fisher’s exact test p-value from comparison of categorical variables LINE-1 methylation in tertiles (<56.7%, 56.7–58.6%, and >58.6%) and SNPs modeled according to codominant mode of in heritance coded as "0" for wild type, "1" heterozygous variant, "2" for homozygous variant.

**Supplemental Material, Table S4. Distributional characteristics of study subjects and LINE-1 methylation in the SBC/EPICURO Study**

| Variables | N | Percent | LINE-1 methylation (%) Median (IQR) | P-value[a] |
|---|---|---|---|---|
| **Age (years)** | | | | |
| <60 | 253 | 28.4 | 57.4 (3.7) | 0.9 |
| 60-69 | 342 | 38.3 | 57.4 (3.2) | |
| 70+ | 297 | 33.3 | 57.5 (3.2) | |
| **Gender** | | | | |
| Male | 792 | 88.8 | 57.5 (3.3) | 0.04 |
| Female | 100 | 11.2 | 57.1 (3.2) | |
| **Region** | | | | |
| Barcelona | 168 | 18.8 | 57.5 (3.0) | 0.1 |
| Vallès | 135 | 15.1 | 57.1 (2.6) | |
| Elche | 73 | 8.2 | 57.1 (2.5) | |
| Tenerife | 145 | 16.3 | 57.3 (3.2) | |
| Asturias | 371 | 41.6 | 57.7 (3.9) | |
| **Body mass index (kg/m$^2$)** | | | | |
| <25.0 | 372 | 53.4 | 57.4 (3.3) | 0.9 |
| 25.0-26.99 | 148 | 21.2 | 57.4 (3.7) | |
| 27.0-29.99 | 120 | 17.2 | 57.5 (3.2) | |
| ≥30.0 | 57 | 8.2 | 57.6 (3.5) | |
| Missing data | 195 | | | |
| **Smoking status** | | | | |
| Non-smoker | 255 | 28.7 | 57.4 (3.2) | 0.2 |
| Occasional smoker | 66 | 7.4 | 58.0 (3.3) | |
| Former smoker | 329 | 37.0 | 57.4 (3.3) | |
| Current smoker | 239 | 26.9 | 57.4 (3.7) | |
| Missing data | 3 | | | |
| **Tobacco type** | | | | |
| Non-smoker | 255 | 31.0 | 57.4 (3.2) | 0.2 |
| Blond only | 99 | 12.0 | 57.0 (3.2) | |
| Black only | 219 | 26.5 | 57.7 (3.6) | |
| Blond and black | 154 | 18.7 | 57.3 (3.6) | |
| Unknown | 97 | 11.8 | 57.7 (3.0) | |
| Missing data | 68 | | | |
| **Controls' diagnosis** | | | | |
| Hernia | 332 | 37.2 | 57.4 (3.1) | 0.8 |
| Fracture & Trauma | 263 | 29.5 | 57.5 (4.0) | |
| Hydrocele | 122 | 13.7 | 57.6 (3.3) | |
| Other Abdominal Surgery | 99 | 11.1 | 57.2 (2.9) | |

| Variables | N | Percent | LINE-1 methylation (%) Median (IQR) | P-value[a] |
|---|---|---|---|---|
| Other Diseases | 76 | 8.5 | 57.6 (3.8) | |
| **Vitamin B1 intake (µg/day/kcal)** | | | | |
| < 0.66 | 323 | 50.1 | 57.5 (3.8) | 0.4 |
| ≥0.66 | 322 | 49.9 | 57.4 (3.3) | |
| Missing data | 247 | | | |
| **Vitamin B2 intake (µg/day/kcal)** | | | | |
| < 0.91 | 323 | 50.1 | 57.4 (3.7) | 0.9 |
| ≥0.91 | 322 | 49.9 | 57.5 (3.6) | |
| Missing Data | 247 | | | |
| **Vitamin B3 intake (µg/day/kcal)** | | | | |
| <10.08 | 323 | 50.1 | 57.5 (3.9) | 0.5 |
| ≥10.08 | 322 | 49.9 | 57.4 (3.3) | |
| Missing Data | 247 | | | |
| **Vitamin B6 intake (µg/day/kcal)** | | | | |
| <1.00 | 323 | 50.1 | 57.4 (3.9) | 0.5 |
| ≥1.00 | 322 | 49.9 | 57.4 (3.4) | |
| Missing Data | 247 | | | |
| **Vitamin B12 intake (µg/day/kcal)** | | | | |
| <3.98 | 323 | 50.1 | 57.4 (3.7) | 0.8 |
| ≥3.98 | 322 | 49.9 | 57.4 (3.6) | |
| Missing Data | 247 | | | |
| **Folate intake (µg/day/kcal)** | | | | |
| <166.74 | 323 | 50.1 | 57.4 (3.9) | 0.5 |
| ≥166.74 | 322 | 49.9 | 57.5 (3.3) | |
| Missing Data | 247 | | | |
| **Protein intake (µg/day/kcal)** | | | | |
| <46.68 | 323 | 50.1 | 57.4 (3.7) | 0.97 |
| ≥4.33 | 322 | 49.9 | 57.4 (3.6) | |
| Missing Data | 247 | | | |
| **Alcohol intake (µg/day/kcal)** | | | | |
| <4.33 | 323 | 50.1 | 57.4 (3.6) | 0.4 |
| ≥4.33 | 322 | 49.9 | 57.4 (3.8) | |
| Missing Data | 247 | | | |
| **Fruit intake (g/day/kcal)** | | | | |
| <172.71 | 320 | 50.1 | 57.3 (3.2) | 0.6 |
| ≥172.71 | 319 | 49.9 | 57.6 (4.2) | |
| Missing Data | 253 | | | |
| **Vegetable intake (g/day/kcal)** | | | | |
| <107.12 | 320 | 50.0 | 57.3 (4.0) | 0.3 |

| Variables | N | Percent | LINE-1 methylation (%) Median (IQR) | P-value[a] |
|---|---|---|---|---|
| ≥107.12 | 320 | 50.0 | 57.6 (3.3) | |
| Missing Data | 252 | | | |
| **Fruit and vegetable intake (g/day/kcal)** | | | | |
| <295.61 | 320 | 50.1 | 57.4 (3.5) | 0.9 |
| ≥295.61 | 319 | 49.9 | 57.4 (3.9) | |
| Missing Data | 253 | | | |
| **Toenail aluminum (μg/g)** | | | | |
| < 10.51 | 329 | 50.0 | 57.5 (3.6) | 0.5 |
| ≥ 10.51 | 329 | 50.0 | 57.4 (3.3) | |
| Missing Data | 234 | | | |
| **Toenail arsenic (μg/g)** | | | | |
| <0.07 | 330 | 50.1 | 57.5 (3.8) | 0.7 |
| ≥0.07 | 329 | 49.9 | 57.4 (3.3) | |
| Missing Data | 233 | | | |
| **Toenail cadmium (μg/g)** | | | | |
| <0.01 | 330 | 50.1 | 57.5 (3.2) | 0.4 |
| ≥0.01 | 329 | 49.9 | 57.4 (3.6) | |
| Missing Data | 233 | | | |
| **Toenail chromium (μg/g)** | | | | |
| < 0.41 | 330 | 50.1 | 57.5 (3.8) | 0.6 |
| ≥ 0.41 | 329 | 49.9 | 57.4 (3.2) | |
| Missing Data | 233 | | | |
| **Toenail copper (μg/g)** | | | | |
| < 3.38 | 329 | 49.9 | 57.5 (3.3) | 0.9 |
| ≥3.38 | 330 | 50.1 | 57.4 (3.5) | |
| Missing Data | 233 | | | |
| **Toenail iron (μg/g)** | | | | |
| < 14.61 | 329 | 50.0 | 57.4 (3.1) | 0.3 |
| ≥ 14.61 | 329 | 50.0 | 57.6 (3.7) | |
| Missing Data | 234 | | | |
| **Toenail lead (μg/g)** | | | | |
| <0.40 | 329 | 50.1 | 57.5 (3.1) | 0.7 |
| ≥0.40 | 330 | 49.9 | 57.4 (3.6) | |
| Missing Data | 233 | | | |
| **Toenail manganese (μg/g)** | | | | |
| < 0.33 | 330 | 50.1 | 57.4 (3.3) | 0.9 |
| ≥ 0.33 | 329 | 49.9 | 57.5 (3.6) | |
| Missing Data | 233 | | | |
| **Toenail nickel (μg/g)** | | | | |

| Variables | N | Percent | LINE-1 methylation (%) Median (IQR) | P-value[a] |
|---|---|---|---|---|
| < 0.47 | 329 | 49.9 | 57.5 (3.0) | 0.8 |
| ≥ 0.47 | 330 | 50.1 | 57.4 (3.9) | |
| Missing Data | 233 | | | |
| **Toenail selenium (μg/g)** | | | | |
| <0.56 | 330 | 50.1 | 57.4 (3.4) | 0.6 |
| ≥0.56 | 329 | 49.9 | 57.5 (3.5) | |
| Missing Data | 233 | | | |
| **Toenail vanadium (μg/g)** | | | | |
| < 0.02 | 326 | 50.1 | 57.5 (3.7) | 0.6 |
| ≥ 0.02 | 325 | 49.9 | 57.4 (3.3) | |
| Missing Data | 241 | | | |
| **Toenail zinc (μg/g)** | | | | |
| < 103.20 | 329 | 49.9 | 57.5 (3.3) | 0.8 |
| ≥103.20 | 330 | 50.1 | 57.4 (3.7) | |
| Missing data | 233 | | | |
| ***NAT2* phenotype** | | | | |
| Rapid/Intermediate acetylator | 389 | 43.9 | 57.4 (3.5) | 0.8 |
| Slow acetylator | 498 | 56.1 | 57.5 (3.2) | |
| Missing data | 5 | | | |
| ***GSTM1* genotype** | | | | |
| (+/+, +/-) | 421 | 47.7 | 57.4 (3.5) | 0.6 |
| (-/-) | 462 | 52.3 | 57.5 (3.1) | |
| Missing data | 9 | | | |
| ***GSTT1* genotype** | | | | |
| (+/+, +/-) | 688 | 77.7 | 57.5 (3.3) | 0.2 |
| (-/-) | 198 | 22.3 | 57.4 (3.3) | |
| Missing data | 6 | | | |

IQR, interquartile range.
[a]P-value from the Kruskal-Wallis test.

**Supplemental Material, Table S5. Association between LINE-1 methylation and individual characteristics of study subjects in the SBC/EPICURO Study**

| Variables | N | β[a] (95% CI) | P-value |
|---|---|---|---|
| **Age (years)** | 892 | -0.004 (-0.02, 0.01) | 0.6 |
| **Gender** | | | |
| Male | 792 | Ref | |
| Female | 100 | -0.5 (-1.0, 0.04) | 0.07 |
| **Region** | | | |
| Barcelona | 168 | Ref | |
| Vallès | 135 | -0.2 (-0.7, 0.4) | 0.6 |
| Elche | 73 | -0.5 (-1.1, 0.2) | 0.2 |
| Tenerife | 145 | -0.1 (-0.7, 0.4) | 0.6 |
| Asturias | 371 | 0.2 (-0.3, 0.6) | 0.5 |
| **Body mass index (kg/m²)** | | | |
| <25.0 | 372 | Ref | |
| 25.0-26.99 | 148 | -0.1 (-0.6, 0.3) | 0.5 |
| 27.0-29.99 | 120 | 0.2 (-0.3, 0.7) | 0.3 |
| ≥30.0 | 57 | 0.007 (-0.7, 0.7) | 0.9 |
| Missing data | 195 | | |
| **Controls' diagnosis** | | | |
| Hernia | 332 | Ref | |
| Fracture & Trauma | 263 | -0.04 (-0.5, 0.4) | 0.8 |
| Hydrocele | 122 | 0.2 (-0.4, 0.7) | 0.5 |
| Other Abdominal Surgery | 99 | 0.1 (-0.7, 0.4) | 0.6 |
| Other Diseases | 76 | 0.2 (-0.4, 0.8) | 0.6 |
| **Dietary intake[b]** | | | |
| Vitamin B1 (µg/day/kcal) | 645 | 0.6 (-0.5, 1.8) | 0.3 |
| Vitamin B2 (µg/day/kcal) | 645 | 0.2 (-0.5, 0.9) | 0.6 |
| Vitamin B3 (µg/day/kcal) | 645 | 0.03 (-0.04, 0.1) | 0.4 |
| Vitamin B6 (µg/day/kcal) | 645 | 0.8 (0.01, 1.7) | 0.05 |
| Vitamin B12 (µg/day/kcal) | 645 | -0.03 (-0.08, 0.02) | 0.2 |
| Folate (µg/day/kcal) | 645 | 0.002 (-0.001, 0.01) | 0.1 |
| Protein (µg/day/kcal) | 645 | 0.01 (-0.01, 0.03) | 0.3 |
| Alcohol (µg/day/kcal) | 645 | -0.01 (-0.03, 0.02) | 0.5 |
| Fruit (g/day/kcal) | 639 | 0.0001 (-0.001, 0.002) | 0.9 |
| Vegetable (g/day/kcal) | 640 | 0.002 (-0.0004, 0.004) | 0.1 |
| Fruit and vegetable (g/day/kcal) | 639 | 0.001 (-0.001, 0.002) | 0.4 |
| **Toenail trace elements[c]** | | | |
| Aluminium (µg/g) | 658 | -0.003 (-0.01, 0.002) | 0.2 |
| Arsenic (µg/g) | 659 | -3.3 (-5.7, -1.0) | 0.006 |
| Cadmium (µg/g) | 659 | 0.1 (-0.3, 0.6) | 0.7 |

| Variables | N | β[a] (95% CI) | P-value |
|---|---|---|---|
| Chromium (µg/g) | 659 | -0.01 (-0.05, 0.03) | 0.7 |
| Copper (µg/g) | 659 | -0.01 (-0.06, 0.05) | 0.8 |
| Iron (µg/g) | 658 | 0.002 (0.001, 0.004) | 0.008 |
| Lead (µg/g) | 659 | -0.05 (-0.1, 0.03) | 0.2 |
| Manganese (µg/g) | 659 | -0.05 (-0.2, 0.06) | 0.4 |
| Nickel (µg/g) | 659 | 0.02 (0.01, 0.03) | 0.004 |
| Selenium (µg/g) | 659 | 0.4 (-0.6, 1.3) | 0.4 |
| Vanadium (µg/g) | 651 | -0.8 (-2.9, 1.3) | 0.4 |
| Zinc (µg/g) | 659 | -0.001 (-0.004, 0.001) | 0.4 |
| ***NAT2* phenotype** | | | |
| Rapid/Intermediate | 389 | Ref | |
| Slow | 498 | 0.2 (-0.1, 0.5) | 0.2 |
| Missing data | 5 | | |
| ***GSTM1* genotype** | | | |
| (+/+, +/-) | 421 | Ref | |
| (-/-) | 462 | 0.02 (-0.3, 0.3) | 0.9 |
| Missing data | 9 | | |
| ***GSTT1* genotype** | | | |
| (+/+, +/-) | 688 | Ref | |
| (-/-) | 198 | -0.2 (-0.6, 0.2) | 0.4 |
| Missing data | 6 | | |

[a]Adjusted for age, gender, and region.
[b]Data available for those who completed food frequency questionnaire.
[c]Data available for those who provided toe nail for trace element assessment.
Note: the exposure contrast for trace elements is 1-µg/g and for dietary variables is 1-µg/day/kcal.

**Supplemental Material, Table S6. Association between LINE-1 methylation and 15 single nucleotide polymorphisms in genes involved in the one-carbon metabolism pathway with nonsignificant results.**

This table is provided as a separate Excel file.

**Supplemental Material, Table S7.** Multivariable model of association between LINE-1 methylation and individual characteristics, toenail trace elements and single nucleotide polymorphisms among study subjects in the SBC/EPICURO Study

| Variable | N | β[a] (95% CI) | P-value | Global p-value[b] |
|---|---|---|---|---|
| **Age (years)** | 601 | -0.01 (-0.03, 0.01) | 0.2 | |
| **Gender** | | | | |
| Male | 534 | Ref | | |
| Female | 67 | -1.0 (-1.7, -0.3) | 0.006 | |
| **Region** | | | | |
| Barcelona | 131 | Ref | | |
| Valles | 90 | -0.05 (-0.7, 0.6) | 0.9 | |
| Elche | 57 | -0.4 (-1.2, 0.3) | 0.3 | |
| Tenerife | 82 | 0.2 (-0.5, 0.9) | 0.5 | |
| Asturias | 241 | 0.1 (-0.4, 0.7) | 0.6 | |
| **Tobacco Type** | | | | |
| Non-smoker | 187 | Ref | | |
| Blond only | 76 | -0.5 (-1.2, 0.2) | 0.1 | |
| Black only | 156 | 0.09 (-0.5, 0.7) | 0.7 | |
| Blond and black | 118 | -0.6 (-1.2, 0.05) | 0.07 | |
| Unknown | 64 | -0.07 (-0.8, 0.7) | 0.9 | |
| **Toenail Trace Element** | | | | |
| Arsenic (µg/g) | 601 | -4.8 (-7.8, -1.8) | 0.002 | |
| Iron (µg/g) | 601 | 0.003 (0.001, 0.01) | 0.0004 | |
| Nickel (µg/g) | 601 | 0.01 (0.004, 0.03) | 0.005 | |
| **Gene, dbSNP identifier** | | | | |
| ***DNMT3A*-rs7581217,** C>T | 601 | 0.3 (0.003, 0.6) | 0.048 | |
| ***AS3MT*-rs7085104** | | | | |
| AA/AG | 518 | Ref | | |
| GG | 83 | 0.5 (-0.07, 1.1) | 0.09 | |
| ***MTHFS*-rs1380642** | | | | |
| CC | 409 | Ref | | |
| CT | 167 | 0.4 (-0.02, 0.9) | 0.06 | 0.005 |
| TT | 25 | -1.2 (-2.2, -0.2) | 0.02 | |
| ***SLC19A1*-rs914238** | | | | |
| TT | 159 | Ref | | |
| TC | 293 | 0.3 (-0.1, 0.8) | 0.2 | 0.07 |
| CC | 149 | -0.2 (-0.8, 0.3) | 0.5 | |
| ***TCN2*-rs9621049** | | | | |
| CC/CT | 593 | Ref | | |
| TT | 8 | 4.3 (0.9, 7.6) | 0.01 | |
| ***TCN2*-rs9606756** | | | | |
| AA/AG | 594 | Ref | | |
| GG | 7 | -2.3 (-7.1, 2.5) | 0.3 | |
| ***TCN2*-rs4820887** | | | | |
| GG/GA | 595 | Ref | | |
| AA | 6 | -0.6 (-6.7, 5.6) | 0.9 | |

[a]Fully adjusted robust regression coefficient.
[b]Global p-value was estimated by using a two-degrees of freedom likelihood-ratio test.

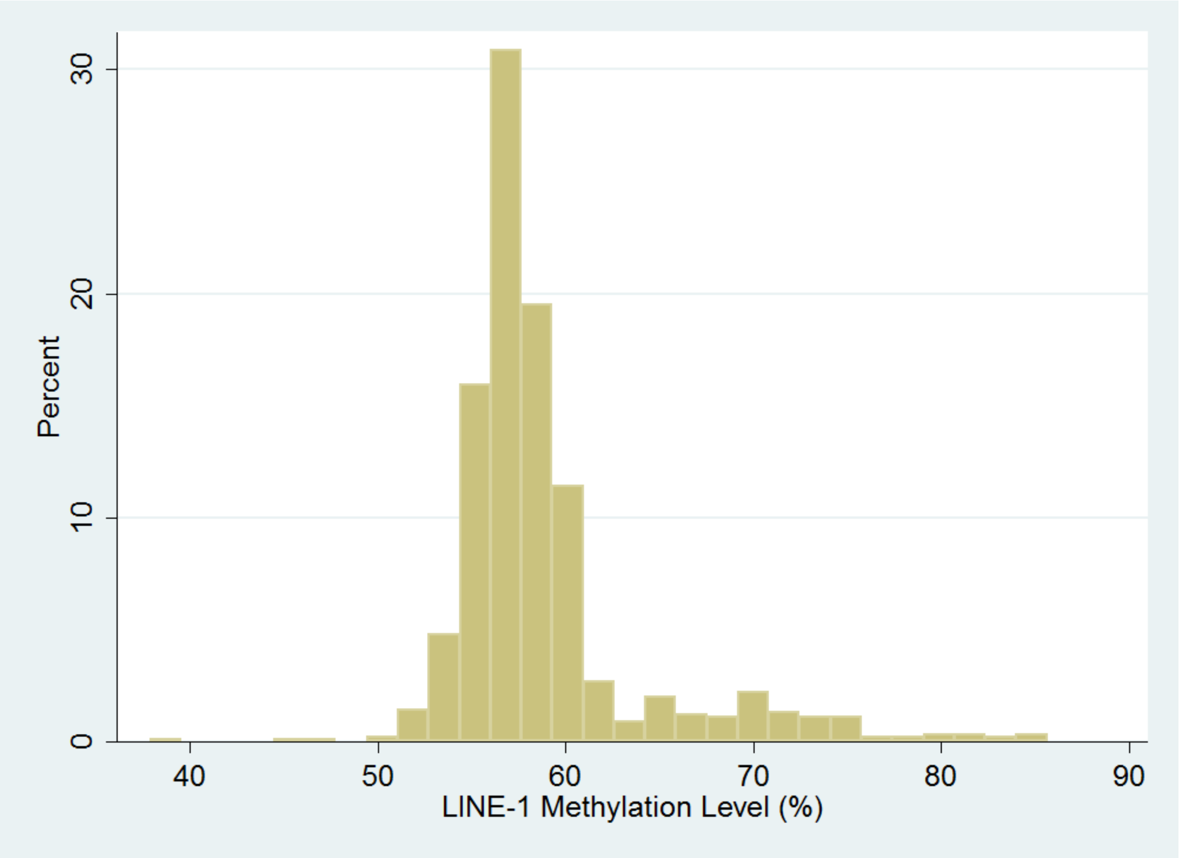


**Supplemental Material, Figure S1. Distribution of LINE-1 methylation among study subjects in the SBC/EPICURO study.**
